# Clinical subtypes of older adults starting long-term care in Japan and their association with prognoses: a data-driven cluster analysis

**DOI:** 10.1038/s41598-024-65699-6

**Published:** 2024-06-28

**Authors:** Yuji Ito, Masao Iwagami, Jun Komiyama, Yoko Hamasaki, Naoaki Kuroda, Ai Suzuki, Tomoko Ito, Tadahiro Goto, Eric Y. F. Wan, Francisco T. T. Lai, Nanako Tamiya

**Affiliations:** 1 Department of General Internal Medicine, Chutoen General Medical Center, Kakegawa, Shizuoka Japan; 2 Health Services Research and Development Center, University of Tsukuba, Tsukuba, Ibaraki Japan; 3 Department of Health Services Research, Graduate School of Comprehensive Human Sciences, University of Tsukuba, Tsukuba, Ibaraki Japan; 4 Health Department, Tsukuba City, Ibaraki Japan; 5 Department of Public Mental Health Research, National Institute of Mental Health, National Center of Neurology and Psychiatry, Tokyo, Japan; 6 Department of Nursing, Institute of Medicine, University of Tsukuba, Tsukuba, Ibaraki Japan; 7 TXP Research, TXP Medical. Co. Ltd., Tokyo, Japan; 8 Department of Family Medicine and Primary Care, School of Clinical Medicine, Li Ka Shing Faculty of Medicine, The University of Hong Kong, Pok Fu Lam, Hong Kong; 9 Department of Pharmacology and Pharmacy, Li Ka Shing Faculty of Medicine, The University of Hong Kong, Pok Fu Lam, Hong Kong; 10 Advanced Data Analytics for Medical Science (ADAMS) Limited, Hong Kong, China; 11 Laboratory of Data Discovery for Health (D24H), Hong Kong Science Park, Sha Tin, Hong Kong

**Keywords:** Long-term care, Multimorbidity, Machine learning, Clustering, Geriatrics, Public health

## Abstract

We aimed to identify the clinical subtypes in individuals starting long-term care in Japan and examined their association with prognoses. Using linked medical insurance claims data and survey data for care-need certification in a large city, we identified participants who started long-term care. Grouping them based on 22 diseases recorded in the past 6 months using fuzzy c-means clustering, we examined the longitudinal association between clusters and death or care-need level deterioration within 2 years. We analyzed 4,648 participants (median age 83 [interquartile range 78–88] years, female 60.4%) between October 2014 and March 2019 and categorized them into (i) musculoskeletal and sensory, (ii) cardiac, (iii) neurological, (iv) respiratory and cancer, (v) insulin-dependent diabetes, and (vi) unspecified subtypes. The results of clustering were replicated in another city. Compared with the musculoskeletal and sensory subtype, the adjusted hazard ratio (95% confidence interval) for death was 1.22 (1.05–1.42), 1.81 (1.54–2.13), and 1.21 (1.00–1.46) for the cardiac, respiratory and cancer, and insulin-dependent diabetes subtypes, respectively. The care-need levels more likely worsened in the cardiac, respiratory and cancer, and unspecified subtypes than in the musculoskeletal and sensory subtype. In conclusion, distinct clinical subtypes exist among individuals initiating long-term care.

## Introduction

With the increase in global aging, the population of older adults requiring long-term care is increasing. In response, several countries, including Japan, South Korea, and Germany, have established public long-term care systems^1^. Japan introduced its public long-term care insurance system in 2000, allowing individuals aged ≥ 65 years, as well as those aged 40–64 years with specified diseases (e.g., stroke), to access long-term care services at home or in facilities when needed^2,3^.

Older adults requiring long-term care tend to have various diseases. In a study by Iwagami et al., which utilized linked medical and long-term care insurance claims data, numerous medical conditions were found to be significantly associated with the initiation of long-term care, with varying degrees of association^4^. The largest odds ratio in the case–control study was found for femoral fractures, followed by dementia, pneumonia, hemorrhagic stroke, and Parkinson’s disease^4^. However, the study examined only the independent association between each disease and the initiation of long-term care and did not evaluate the patterns of multiple diseases or multimorbidity.

Older patients often present with multiple underlying diseases. Mitsutake et al. revealed that approximately 80% of older patients (aged ≥ 75 years) in Tokyo had two or more comorbidities, with several patterns identified^5^. However, this study did not specifically address individuals requiring long-term care, and it did not investigate the association between disease patterns and patient prognosis. Some researchers have examined the association between clinical subtypes and prognosis in the general older population using machine learning methods^6,7^. However, to the best of our knowledge, no study has investigated this association among older adults starting long-term care, a growing population worldwide.

Applying an unsupervised machine-learning approach (to prevent preconceptions) to linked medical and long-term care datasets, this study aimed to identify the clinical subtypes of older adults starting long-term care and examine the longitudinal associations between different clinical subtypes and prognoses.

## Results

### Study participants and their comorbidities

**Figure 1:**
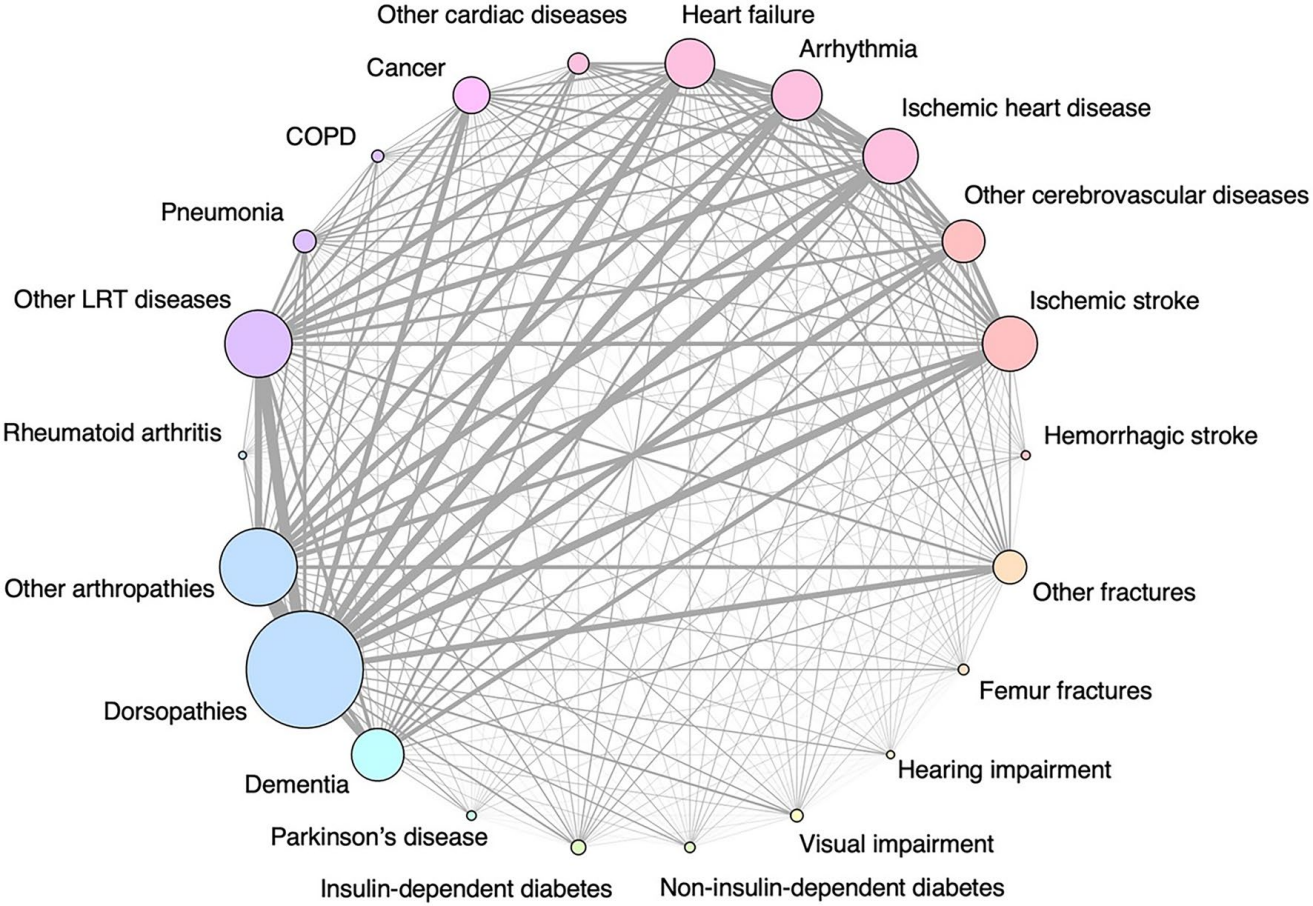
Network plot for each comorbidity in the overall population. *COPD* chronic obstructive pulmonary disease, *Other LRT diseases* other lower respiratory tract diseases. Each circle size represents the number of participants with that disease. The width of each connected line represents the number of participants with both the diseases.

In Tsukuba City, Ibaraki Prefecture, Japan, among the 4831 participants meeting the inclusion criteria, we excluded 151 aged < 65 years and 32 who died before starting long-term care services; the remaining 4648 participants were included in the analysis. The median age of the participants was 83 (interquartile range [IQR], 78–88) years, and 60.4% were females. The median number of comorbidities was 4 (IQR, 3–6), and 4118 (88.6%) participants had two or more comorbidities. The most frequently observed disease in the final sample was dorsopathies (n = 2777, 59.8%), followed by other arthropathies (n = 1840, 39.6%). The relationship between each disease is shown in Fig. 1.

### Clustering the study participants based on the comorbidities

**Figure 2:**
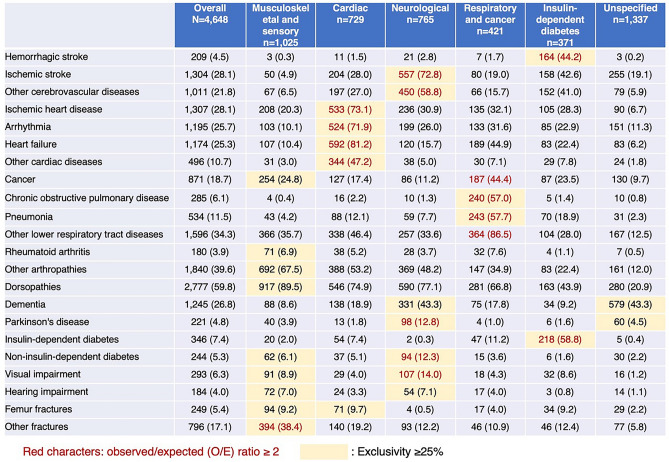
Prevalence of each disease and results of clustering analysis. We highlighted the unique characteristic of each clinical subtype based on an observed/expected ratio ≥ 2 (red characters) and exclusivity ≥ 25% (yellow cells) in line with those in previous studies^7,28^.

For fuzzy c-means clustering, we determined that the optimal number of clusters was six according to the results of the elbow method and the Xie-Beni index (see Supplementary Fig. S1 and Supplementary Table S1 online). We identified six clinical subtypes and named them musculoskeletal and sensory (n = 1025, 22.1%), cardiac (n = 729, 15.7%), neurological (n = 765, 16.5%), respiratory and cancer (n = 421, 9.1%), insulin-dependent diabetes (n = 371, 8.0%), and unspecified (n = 1337, 28.8%) according to the diseases included in each subtype (Fig. 2). The baseline characteristics of the participants are shown in Table 1.

**Table 1:** Characteristics of overall participants and each subtype.

	Overall N = 4648	Musculoskeletal and sensory n = 1025	Cardiac n = 729	Neurological n = 765	Respiratory and cancer n = 421	Insulin-dependent diabetes n = 371	Unspecified n = 1337	P-value
Age, median (IQR)	83 (78–88)	84 (79–88)	85 (81–89)	83 (78–87)	83 (78–88)	80 (73–84)	82 (76–87)	< 0.001
Age category, n (%)								< 0.001
65–69	258 (5.6)	42 (4.1)	14 (1.9)	25 (3.3)	19 (4.5)	56 (15.1)	102 (7.6)	
70–74	476 (10.2)	81 (7.9)	51 (7.0)	80 (10.5)	43 (10.2)	61 (16.4)	160 (12.0)	
75–79	721 (15.5)	142 (13.9)	82 (11.3)	133 (17.4)	59 (14.0)	66 (17.8)	239 (17.9)	
80–84	1252 (26.9)	283 (27.6)	197 (27.0)	230 (30.1)	117 (27.8)	97 (26.2)	328 (24.5)	
85–89	1,200 (25.8)	292 (28.5)	236 (32.4)	194 (25.4)	103 (24.5)	67 (18.1)	308 (23.0)	
≥ 90	741 (15.9)	185 (18.1)	149 (20.4)	103 (13.5)	80 (19.0)	24 (6.5)	200 (15.0)	
Sex females, n (%)	2809 (60.4)	777 (75.8)	457 (62.7)	438 (57.3)	151 (35.9)	171 (46.1)	815 (61.0)	<0.001
Initial long-term care need levels, n (%)								< 0.001
Care need level 1	2399 (51.6)	547 (53.4)	339 (46.5)	411 (53.7)	171 (40.6)	130 (35.0)	801 (59.9)	
Care need level 2	1165 (25.1)	279 (27.2)	205 (28.1)	193 (25.2)	106 (25.2)	78 (21.0)	304 (22.7)	
Care need level 3	525 (11.3)	111 (10.8)	104 (14.3)	81 (10.6)	67 (15.9)	52 (14.0)	110 (8.2)	
Care need level 4	415 (8.9)	72 (7.0)	59 (8.1)	61 (8.0)	57 (13.5)	78 (21.0)	88 (6.6)	
Care need level 5	144 (3.1)	16 (1.6)	22 (3.0)	19 (2.5)	20 (4.8)	33 (8.9)	34 (2.5)	
No. of comorbidities, median (IQR)	4 (3–6)	3 (3–4)	6 (5–7)	5 (4–6)	5 (4–7)	4 (3–6)	2 (1–2)	< 0.001

*IQR* interquartile range.

In the validation analysis in another city (Sammu City, Chiba Prefecture, Japan), the results of clustering were generally similar: comorbidities of 1,628 participants were classified into musculoskeletal and sensory (n = 335, 20.6%), cardiac (n = 196, 12.0%), neurological (n = 224, 13.8%), respiratory and cancer (n = 282, 17.3%), insulin-dependent diabetes (n = 208, 12.8%), and unspecified subtypes (n = 383, 23.5%) (see Supplementary Fig. S2 online).

### Longitudinal analysis between the clinical subtypes and death

**Figure 3:**
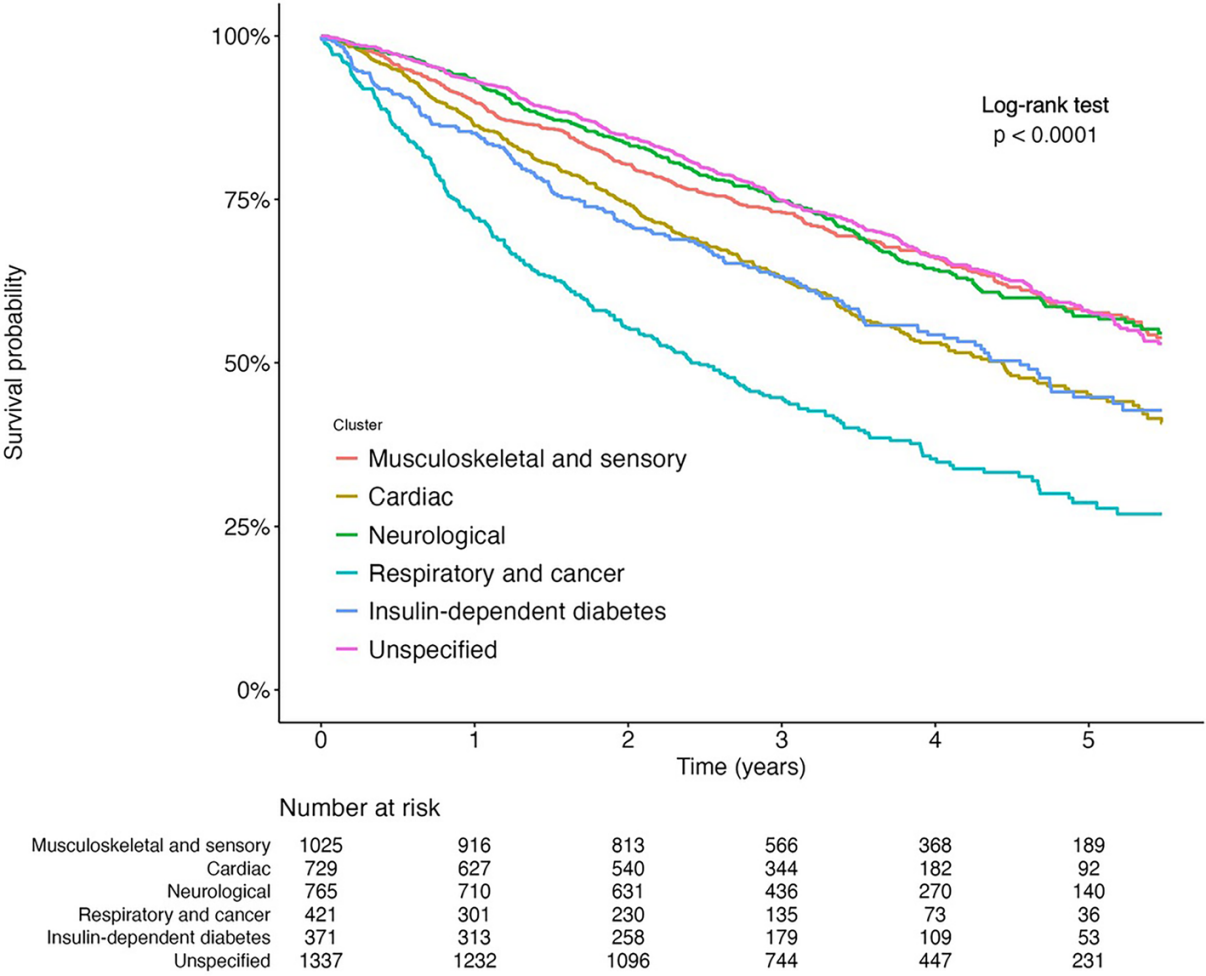
Kaplan–Meier survival curves for each subtype.

Table 2 shows the results of the cohort analysis in Tsukuba City. After a median follow-up of 36.8 (IQR 24.8–52.5) months, 1879 deaths occurred. Figure 3 shows Kaplan–Meier survival curves for overall survival across clinical subtypes, whereas Supplementary Fig. S3 shows the Nelson-Aalen cumulative hazard estimates, which roughly support the proportional hazards assumptions. Compared with musculoskeletal and sensory subtype, the adjusted hazard ratio (95% confidence interval [CI]) for death was 1.22 (1.05–1.42), 0.86 (0.74–1.01), 1.81 (1.54–2.13), 1.21 (1.00–1.46), and 0.90 (0.78–1.03) for the cardiac, neurological, respiratory and cancer, insulin-dependent diabetes, and unspecified subtypes, respectively (Table 2).

**Table 2:** Details of longitudinal analysis and results of Cox regression analysis for death.

	Musculoskeletal and sensory n = 1025	Cardiac n = 729	Neurological n = 765	Respiratory and cancer n = 421	Insulin-dependent diabetes n = 371	Unspecified n = 1337
Death, n (%)	368 (35.9)	338 (46.6)	272 (35.6)	268 (63.7)	175 (47.2)	458 (34.3)
Follow-up months, median (IQR)	38.3 (25.8–55.2)	34.8 (23.1–47.6)	39.4 (27.8–54.8)	26.3 (10.3–41.8)	35.1 (18.0–51.1)	38.2 (27.3–53.8)
Death, per 100 person-year (95%CI)	10.9 (9.8–12.0)	15.7 (14.1–17.4)	10.5 (9.3–11.9)	27.1 (24.0–30.5)	16.0 (13.8–18.5)	10.3 (9.4–11.3)
Cox regression analysis						
Unadjusted hazard ratio (95% CI)	1 (reference)	1.46 (1.26–1.69)	0.97 (0.83–1.13)	2.55 (2.17–2.98)	1.48 (1.24–1.77)	0.95 (0.83–1.09)
Adjusted* hazard ratio (95% CI)	1 (reference)	1.22 (1.05–1.42)	0.86 (0.74–1.01)	1.81 (1.54–2.13)	1.21 (1.00–1.46)	0.90 (0.78–1.03)

*CI* confidence interval, *IQR* interquartile range.

*Adjusted for age, sex, and the initial care-need level.

### Longitudinal analysis between the clinical subtypes and deterioration of care-need levels within 2 years

Table 3 shows the change in care-need levels within 2 years for each subtype. In the multivariable logistic regression analysis adjusted for age, sex, and the initial care-need level, the cardiac (adjusted odds ratio [aOR] 1.39, 95% CI 1.08–1.80), respiratory and cancer (aOR 2.29, 95% CI 1.67–3.15), and unspecified subtypes (aOR 1.47, 95% CI 1.18–1.83) were significantly associated with an increased risk of care-need level deterioration (including death) within 2 years compared with the musculoskeletal and sensory subtype. In the first sensitivity analysis, which excluded those with no reassessment of care-need levels, the results were generally similar to those in the primary analysis. In both the main analysis and the first sensitivity analysis which accounted for death, the aOR of each subtype, especially for the cardiac subtype, was influenced and possibly mediated by the number of comorbidities. In the second sensitivity analysis excluding those with death, the cardiac (aOR 1.45, 95% CI 1.04–2.02), neurological (aOR 1.66, 95% CI 1.23–2.25), respiratory and cancer (aOR 1.63, 95% CI 1.05–2.53) and unspecified (aOR 2.04, 95% CI 1.57–2.67) subtypes were significantly associated with an increased risk of care-need level deterioration (excluding death), indicating that the results in the primary analysis for the neurological subtype was largely influenced by death.

**Table 3:** Details of changes in care-need levels and results of logistic regression analysis for care-need level deterioration within two years.

	Musculoskeletal and sensory n = 618	Cardiac n = 412	Neurological n = 459	Respiratory and cancer n = 244	Insulin-dependent diabetes n = 214	Unspecified n = 799
Care-need levels reassessed within 2 years						
Improved, n (%)	115 (18.6)	71 (17.2)	60 (13.1)	28 (11.5)	42 (19.6)	87 (10.9)
No change, n (%)	214 (34.6)	116 (28.2)	167 (36.4)	49 (20.1)	62 (29.0)	273 (34.2)
Deteriorated, n (%)	126 (20.4)	96 (23.3)	144 (31.4)	44 (18.0)	35 (16.4)	277 (34.7)
Dead within two years, n (%)	121 (19.6)	112 (27.2)	74 (16.1)	113 (46.3)	58 (27.1)	125 (15.6)
Alive but care-need levels not reassessed within 2 years, n (%)	39 (6.3)	17 (4.1)	13 (2.8)	10 (4.1)	15 (7.0)	33 (4.1)
Loss-to-follow-up at 2 years, n (%)	3 (0.5)	0 (0.0)	1 (0.2)	0 (0.0)	2 (0.9)	4 (0.5)
Logistic regression analysis for care-need level deterioration*						
Unadjusted odds ratio (95% CI)	1 (reference)	1.52 (1.18–1.95)	1.35 (1.06–1.73)	2.69 (1.98–3.66)	1.16 (0.85–1.60)	1.52 (1.23–1.89)
Adjusted odds ratio (95% CI)§	1 (reference)	1.39 (1.08–1.80)	1.27 (0.99–1.63)	2.29 (1.67–3.15)	1.22 (0.87–1.70)	1.47 (1.18–1.83)
Adjusted odds ratio (95% CI)^¶^	1 (reference)	1.17 (0.88–1.55)	1.15 (0.89–1.49)	2.01 (1.44–2.79)	1.15 (0.82–1.60)	1.69 (1.33–2.14)
Sensitivity analysis 1^†^						
Unadjusted odds ratio (95% CI)	1 (reference)	1.48 (1.14–1.92)	1.28 (1.00–1.64)	2.72 (1.97–3.74)	1.19 (0.86–1.65)	1.49 (1.20–1.85)
Adjusted odds ratio (95% CI)§	1 (reference)	1.36 (1.05–1.77)	1.19 (0.92–1.53)	2.33 (1.67–3.23)	1.24 (0.88–1.74)	1.42 (1.13–1.77)
Adjusted odds ratio (95% CI)^¶^	1 (reference)	1.17 (0.88–1.57)	1.10 (0.85–1.43)	2.07 (1.47–2.92)	1.18 (0.83–1.66)	1.60 (1.25–2.04)
Sensitivity analysis 2^‡^						
Unadjusted odds ratio (95% CI)	1 (reference)	1.34 (0.97–1.85)	1.66 (1.24–2.22)	1.49 (0.98–2.28)	0.88 (0.57–1.36)	2.01 (1.55–2.60)
Adjusted odds ratio (95% CI)^§^	1 (reference)	1.45 (1.04–2.02)	1.66 (1.23–2.25)	1.63 (1.05–2.53)	1.20 (0.76–1.90)	2.04 (1.57–2.67)
Adjusted odds ratio (95% CI)^¶^	1 (reference)	1.45 (1.02–2.07)	1.66 (1.22–2.27)	1.63 (1.03–2.57)	1.20 (0.76–1.90)	2.05 (1.52–2.75)

*CI* confidence interval.

*The outcome was “deteriorated” care-need levels or death within 2 years. Those with loss-to-follow-up were excluded from the analysis. Those alive without reassessment of care-need levels within 2 years were not excluded, assuming that their care-need level did not change.

^†^The outcome was “deteriorated” care-need levels or death within 2 years. Those with loss-to-follow-up, and those alive without reassessment of care-need levels within 2 years were excluded from the analysis.

^‡^The outcome of interest was “deteriorated” care-need levels or death within 2 years. Those who died within 2 years, those with loss-to-follow-up, and those alive without reassessment of care-need levels within 2 years were excluded from the analysis.

^§^Adjusted for age, sex, and the initial care-need level.

^¶^Adjusted for age, sex, the initial care-need level, and the number of comorbidities.

## Discussion

In this study, using unsupervised machine learning, we identified six distinct clinical subtypes among individuals aged ≥ 65 years who started long-term care in a city in Japan. The results of clustering were similar to that in another city. In the longitudinal analysis, individuals with cardiac, respiratory and cancer, and insulin-dependent diabetes subtypes had a significantly higher risk of death than those with the musculoskeletal and sensory subtype, independent of age, sex, and initial care-need levels. Furthermore, individuals with cardiac, respiratory and cancer, and unspecified subtypes were at a higher risk of deterioration of the care-need level within 2 years than those with the musculoskeletal and sensory subtype.

Clustering is considered an active method of grouping data into many collections or clusters according to the similarities in data point features and characteristics^8^. By employing machine learning-based clustering methods without predefined hypotheses, we can expand our understanding of the characteristics inherent within a heterogeneous group beyond our initial perceptions^9^. Several studies have applied clustering methods to categorize individuals with a disease or condition into more homogenous subgroups. For example, Cohen et al. identified three clinical phenogroups of heart failure with preserved ejection fraction and their different responses to spironolactone^10^. Ahlqvist et al. identified five replicable clusters among patients with diabetes showcasing different disease progressions and risks of diabetic complications^11^.

The older population is inherently diverse, encompassing a spectrum of comorbidities^5^ and socioeconomic backgrounds. Stratifying a heterogeneous group of individuals requiring long-term care into distinct subtypes can be beneficial for precisely understanding their characteristics and exploring more tailored and effective care approaches. Previous studies using clustering methods revealed that some clinical subtypes of chronic conditions in older adults have synergistic adverse effects^6,7,12^. In a Stockholm-based study, using a fuzzy c-means clustering algorithm, Tazzeo et al. found that older adults with multimorbidity, characterized by metabolic and sleep disorder patterns; cardiovascular, anemia, and dementia patterns; and psychiatric disease patterns were associated more with incident physical frailty in 6 years^7^. Marengoni et al. found that those with cardiovascular, anemia, and dementia patterns were associated with an increased risk of institutionalization in 6 years compared with those with an unspecified pattern^12^. Notably, these findings exhibit substantial differences from the outcomes of this study, possibly due to the different characteristics of the study population in different countries and/or the different factors used for clustering. In contrast, Ibarra-Castillo et al., in a study conducted in Barcelona using a k-means clustering algorithm, delineated six multimorbidity patterns and their associations with mortality^6^. Their patterns encompassed musculoskeletal, endocrine-metabolic, neurological, cardiovascular, digestive-respiratory (in men only), digestive (in women only), and nonspecific patterns. Compared with the musculoskeletal pattern, the other patterns were associated with a significantly higher risk of death. These patterns are similar to those observed in this study.

In our opinion, some potential reasons contributing to each clinical subtype showing different outcomes could be the types and number of comorbidities involved. The three leading causes of death in Japan in 2022 were malignancy, heart disease, and senility^13^. This may explain why the cardiac subtype and the respiratory and cancer subtype had worse mortality outcomes. People with insulin-dependent diabetes may have had longer periods of morbidity and more complications than in people with non-insulin-dependent diabetes. This may explain why the insulin-dependent diabetes subtype had worse mortality. Regarding the deterioration of care-need levels within 2 years, diseases that could progress and affect activities of daily living in the relatively short period (e.g. heart disease and malignancy) may have influenced the results. The neurological subtype with the same prevalence of dementia as in the unspecified subtype had a better outcome, despite having more comorbidities. Therefore, we considered that dementia in the unspecified subtype was not associated with a negative outcome and that there might be another unmeasured risk factor affecting the unspecified subtype.

This study holds several clinical and research implications. In clinical settings, the findings suggest that medical and long-term care staff should be more conscious of the clinical subtypes among long-term care users. This information can be used when engaging in shared decision-making with patients and their family members or caregivers, facilitating better preparation for prognostic considerations. In the realm of research, the current study results can be used for classification or restriction of the study population in future interventional studies to examine the effectiveness of specific interventions. While certain services have demonstrated efficacy in individuals certified as requiring support to prevent the deterioration of care-need levels^14,15^, the study suggests that these services may also prove effective in specific subtypes of individuals certified as needing care. Ultimately, medical and long-term care services should be tailored according to care-need levels and clinical subtypes^16,17^.

This study had some limitations. First, this study was conducted in one city in Japan, and a validation study was conducted in another city. Although the clustering results were generally similar between the two cities, generalizability may still be a concern. Thus, the finding should be replicated in other areas of Japan, as well as in other countries. Second, misclassification of each of the 22 diseases was possible mainly because the disease status was based on the recorded International Classification of Diseases 10th Revision (ICD-10) codes in the medical claims. The results of this study should be replicated in another study with primary data collection, in which the diagnosis is ensured by the responsible physicians. Third, in any studies using clustering, to name each cluster could be considered subjective. This may be true in the present study, and different researchers could name each cluster differently based on their clinical experience. In addition, we conducted this study based solely on 22 diseases listed by the Ministry of Health, Labour and Welfare, which did not include other diseases such as psychiatric disorders and anemia. Considering the potential inclusion of psychiatric disorders^7,18^ in the identified "unspecified" subtype, further research, including potential diseases other than those in this study, is necessary to clarify the characteristics of unspecified subtypes in more detail. Additionally, in the longitudinal analysis, we adjusted for age, sex, and the initial care-need level as potential confounding factors; however, there may be other (unmeasured) confounding factors, such as socioeconomic status.

## Conclusions

We identified novel clinical subtypes of older adults starting long-term care in Japan and found that their association with prognosis differed between the subtypes. The study findings indicated long-term care strategies and informed consent from patients and their family members can be tailored according to the clinical subtypes.

## Methods

### Study design and setting

We conducted both cross-sectional (for clustering at baseline) and longitudinal analyses in a cohort study (for examining the association between clinical subtypes and all-cause mortality and deterioration of care-need levels) using linked medical insurance claims data and survey data for care-need certification in Tsukuba City in Japan (“main analysis”). In addition, we examined whether our clustering approach was replicable in Sammu City in Japan (“validation analysis”).

### Ethical approval

This study was conducted according to the principles of the Declaration of Helsinki. The study was approved and informed consent was waived due to the anonymous nature of the data by the Ethics Committee, Institute of Medicine, University of Tsukuba (approval number: 1445-14).

### Data source

In Japan, each city maintains medical claims data within the Late-stage Elderly Health System (for those aged ≥ 75 years), National Health Insurance system (for those aged < 75 years), and long-term care certification data. Through a joint research contract, we requested Tsukuba city and Sammu city to provide their data. Tsukuba City's data, with its larger sample size, were considered the derivative sample. Sammu City's data served as the validating sample.

For the main analysis, medical claims data (April 2014 to March 2019), survey data for care-need certification (February 2012 to June 2019), and insurance registration data (October 2014 to March 2021) of enrollees of the Late-stage Elderly Health System or National Health Insurance were obtained from the municipal government of Tsukuba City, Ibaraki Prefecture, Japan. Tsukuba City is a large region with a population of 240,383 people, including 46,613 (19.4%) people aged ≥ 65 years^19^.

For the validation analysis, medical claims data (May 2012 to October 2016) and survey data for care-need certification (May 2012 to October 2016) of enrollees of the Late-stage Elderly Health System or National Health Insurance were obtained from the municipal government in Sammu City, Chiba Prefecture, Japan. The city has a population of 48,444, including 17,329 (35.8%) people aged ≥ 65 years^20^. We conducted only the cross-sectional analysis in Sammu City because the follow-up data were not sufficiently available.

The details of the Japanese medical and long-term care insurance claims data are described elsewhere^21–23^. Briefly, the medical insurance claims include medical service fees, dates of receiving medical services, diseases according to the ICD-10 codes, tests conducted, and drugs prescribed. Under the long-term care insurance system, people are certified through a standardized process involving an assessment of physical and cognitive functions^24^ and are categorized into seven grades, as follows: support levels 1 and 2 and care levels from 1 to 5, which are well correlated with the Barthel Index (r = − 0.70)^25^. They receive periodic reassessments of care-need levels, with intervals ranging from 3 to 24 months during our study period^26^.

We integrated the medical insurance claims data and survey data for care-need certification and the insurance registration data in the case of Tsukuba City, using the pseudo-ID provided by the municipality.

### Study population

In Tsukuba City, we identified people receiving the survey for long-term care needs certification for the first time between October 2014 (ensuring a 6-month look-back period after April 2014 for defining comorbidities from medical claims) and March 2019 (see Supplementary Fig. S4 online). The 6-month look-back period was in line with that in our previous study^4^, wherein we assumed that diagnoses associated with the long-term care incidence would be recorded within 6 months before long-term care certification in the Japanese healthcare system. In this study, we included those with care levels ranging from 1 to 5, as individuals with support levels often do not require actual long-term care and instead receive preventive care to forestall the need for long-term care. We excluded individuals aged < 65 years and those who died before starting the actual long-term care services.

In Sammu City, for the validation analysis, we identified the participants between May 2013 and October 2016 and applied the same exclusion criteria.

### Exposures (factors used for clustering)

According to the Ministry of Health, Labour, and Welfare in the Comprehensive Survey of Living Conditions in Japan, 22 diseases are considered to potentially affect the initiation of long-term care^4^. The list includes hemorrhagic stroke, ischemic stroke, other cerebrovascular diseases, ischemic heart disease, arrhythmia, heart failure, other cardiac diseases, cancer, chronic obstructive pulmonary disease, pneumonia, other lower respiratory tract diseases, rheumatoid arthritis, other arthropathies, dorsopathies, dementia, Parkinson's disease, insulin-dependent diabetes, non-insulin-dependent diabetes, visual impairment, hearing impairment, femur fractures, and other fractures.

We defined the presence or absence of each of the 22 diseases, which were recorded in medical claims during the past 6 months before the certification of long-term care (including the same month when the long-term care was certified). We utilized the ICD-10 codes used in previous studies (see Supplementary Table S2 online)^4^.

### Outcomes

In the cohort analysis in Tsukuba City, the outcomes of interest were all-cause mortality and deterioration of care need levels within 2 years. Data on deaths were obtained from insurance registration, a source theoretically capable of capturing all fatalities up to March 2021. Information on care need levels was derived from the survey data for care-need certification. Specifically, we retrieved the certified care-need levels of participants at or closest to the time point 2 years (i.e., 24 months) from the date of initial certification. Our decision of having a 2-year follow-up period was due to the fact the participants starting that long-term care should have had received periodic reassessments of care-need levels at least once in 24 months during our study period, in the Japanese long-term care system^26^. In the primary analysis, deterioration of care-need levels was defined as an increase in the level of the latest certification compared with the initial level, or death within 24 months.

### Statistical analyses

We summarized the characteristics of the study participants using frequencies (percentages) for categorical variables and medians (IQRs) for continuous variables. The prevalence of each disease and the number of comorbidities (among 22 comorbidities) per person were calculated. We constructed a network plot to identify the relationship between each comorbidity.

For clustering, after conducting dimensionality reduction through multiple correspondence analysis using the “FactoMineR” package of R, we employed a fuzzy c-means clustering algorithm using the “e1071” package of R, allowing individuals to belong to more than one cluster. We employed the elbow method and the Xie-Beni index (optimal when presenting low values)^27^ to obtain the optimal cluster number, with various cluster numbers tested. We repeated the fuzzy c-means clustering algorithm 100 times to account for the random nature of the cluster solutions, and an average outcome was generated. Participants were assigned to the cluster with the highest membership probability. The observed/expected ratios of diseases and disease exclusivity were used to describe the disease patterns of the clusters^7,28^. Observed/expected ratios were calculated by dividing the disease prevalence in the cluster by the disease prevalence in the overall population. Disease exclusivity was calculated as the number of participants with a specific disease in a cluster divided by the total number of individuals with that disease. Clusters were characterized based on diseases with an exclusivity of ≥ 25% or an observed/expected ratio of ≥ 2^7,28^. Other basic characteristics of the clusters were compared using the Kruskal–Wallis test for continuous variables, such as age and the number of morbidities, and the chi-square test for categorical variables, such as sex and care need levels.

To validate the replicability of the clustering results, in Sammu City, we repeated the same clustering procedure and then compared the results with those obtained from Tsukuba City.

Finally, each cluster was named based on the clustering results of two cities after discussions among the authors who had clinical experience in treating older patients.

In the cohort analysis in Tsukuba City, Kaplan–Meier survival curves were plotted for death. Additionally, we conducted a Cox proportional hazards analysis for death, with adjustments for age, sex, and initial care-need level. The follow-up continued until the incidence of death, loss to follow-up (defined as removal from the insurance registration for reasons such as moving), or at the end of observation in March 2021. Among the groups except that considered the unspecified subtype, we regarded the group with the largest number of participants as the reference group.

Regarding the deterioration of care-need levels within 2 years, the analysis was restricted to participants who started long-term care before June 2017, so that all the participants (except for those with loss to follow-up) had follow-up information for 2 years. We made a table displaying the number of participants with improved, stable, and deteriorated care-need levels and those with no reassessment, death, or loss to follow-up at 2 years. Excluding those lost to follow-up at 2 years, univariable and multivariable logistic regression analyses were conducted for the deterioration of care-need level (i.e., an increase in the level of the latest certification compared with the initial level or death), with adjustment for age, sex, and the initial care-need level (Model 1) and additionally for the number of comorbidities (Model 2). Care-need level of those with no reassessment was assumed to be unchanged. In the first sensitivity analysis, we excluded those with no reassessment by regarding them as lost to follow-up. In the second sensitivity analysis, individuals with death were further excluded from the analysis, focusing solely on the increase in the latest certification level compared with the initial level.

The significance level was set at p < 0.05. Statistical analyses were performed using R Statistical Software (v4.3.2; R Core Team 2023) and Stata (version 17; StataCorp LLC 2021).

### Supplementary Information

Supplementary Information.

## Data Availability

The data that support the findings of this study are available from Tsukuba City and Sammu City, Japan, but restrictions apply to the availability of these data, which were used under license for the current study, and so are not publicly available. Data are however available from the authors upon reasonable request and with permission of Tsukuba City and Sammu City.
