# Supplementary material for: Clinical subtypes of older adults starting long-term care in Japan and their association with prognoses: a data-driven cluster analysis
Source: Sci Rep. 2024 Jun 28;14:14911. doi: 10.1038/s41598-024-65699-6 (PMC11213852; doi:10.1038/s41598-024-65699-6)
Supplement: Supplementary file 1 — Supplementary Information. [file 41598_2024_65699_MOESM1_ESM.docx]

**Supplementary materials**

**Supplementary Figure S1.** The elbow method for the optimal number of clusters

**Supplementary Figure S2.** Prevalence of each disease and results of clustering analysis as a validation study in Sammu City

**Supplementary Figure S3.** Nelson-Aalen cumulative hazard estimates

**Supplementary Figure S4.** Obtained survey data for care-need certification, medical claims data and insurance registration data in Tsukuba City

**Supplementary Table S1.** The Xie-Beni index of each condition in a fuzzy c-means clustering algorithm

**Supplementary Table S2.** Definition of 22 diseases based on the International Classification of Diseases 10th Revision (ICD-10) codes

**Supplementary Figure S1. The elbow method for the optimal number of clusters**

**
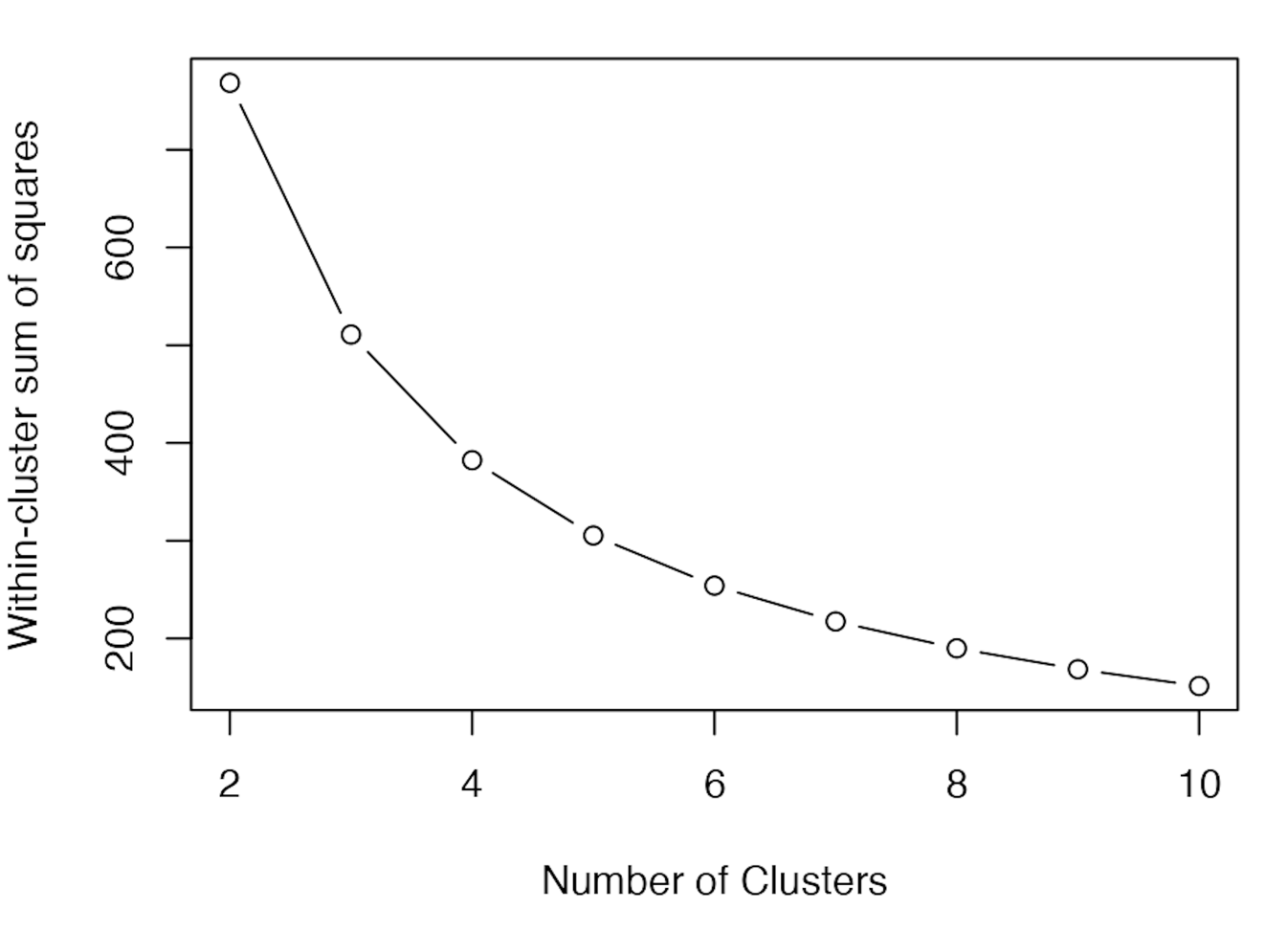
**

**Supplementary Figure S2. Prevalence of each disease and results of clustering analysis as a validation study in Sammu City**


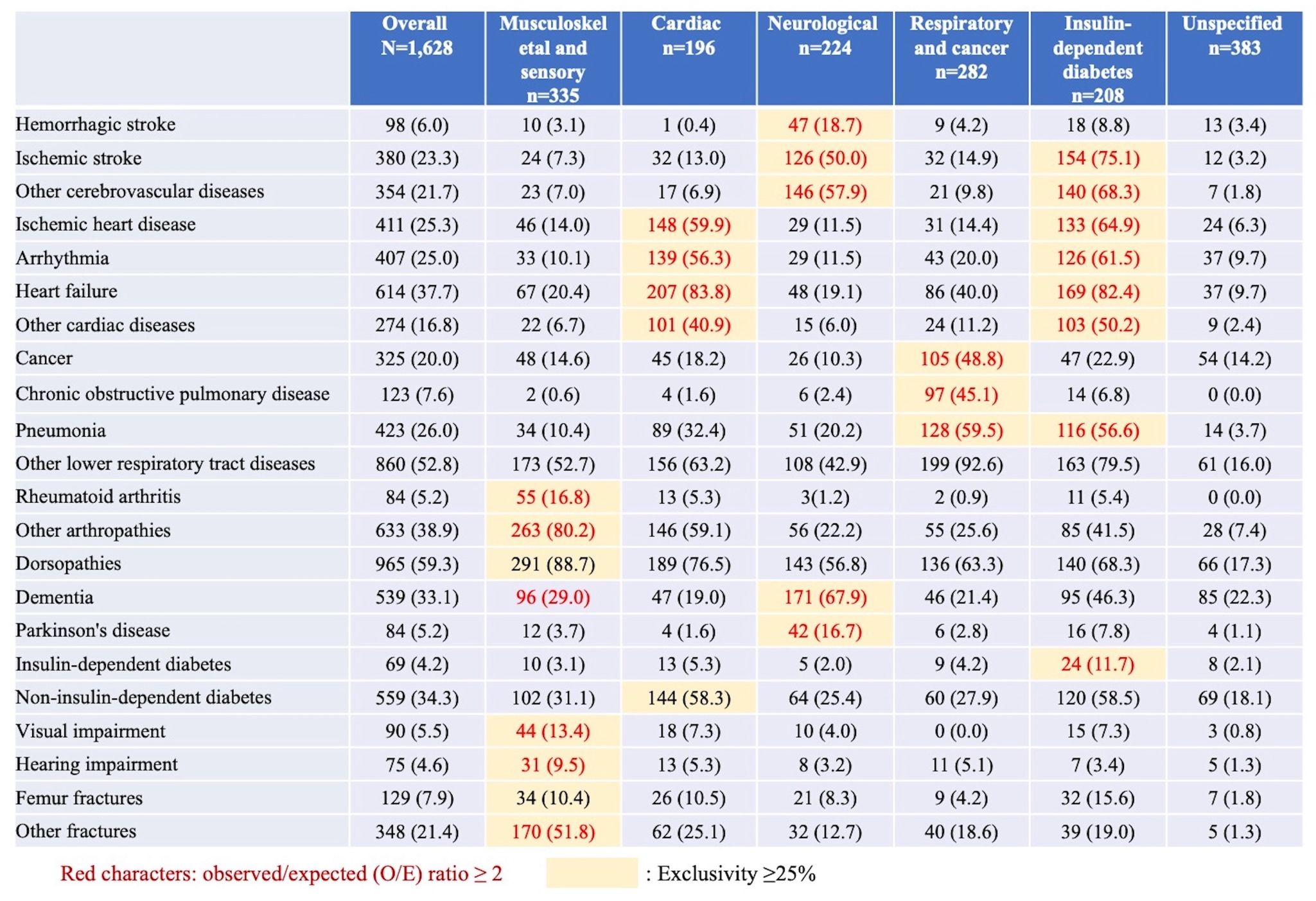


**Supplementary Figure S3.** **Nelson-Aalen cumulative hazard estimates**

**
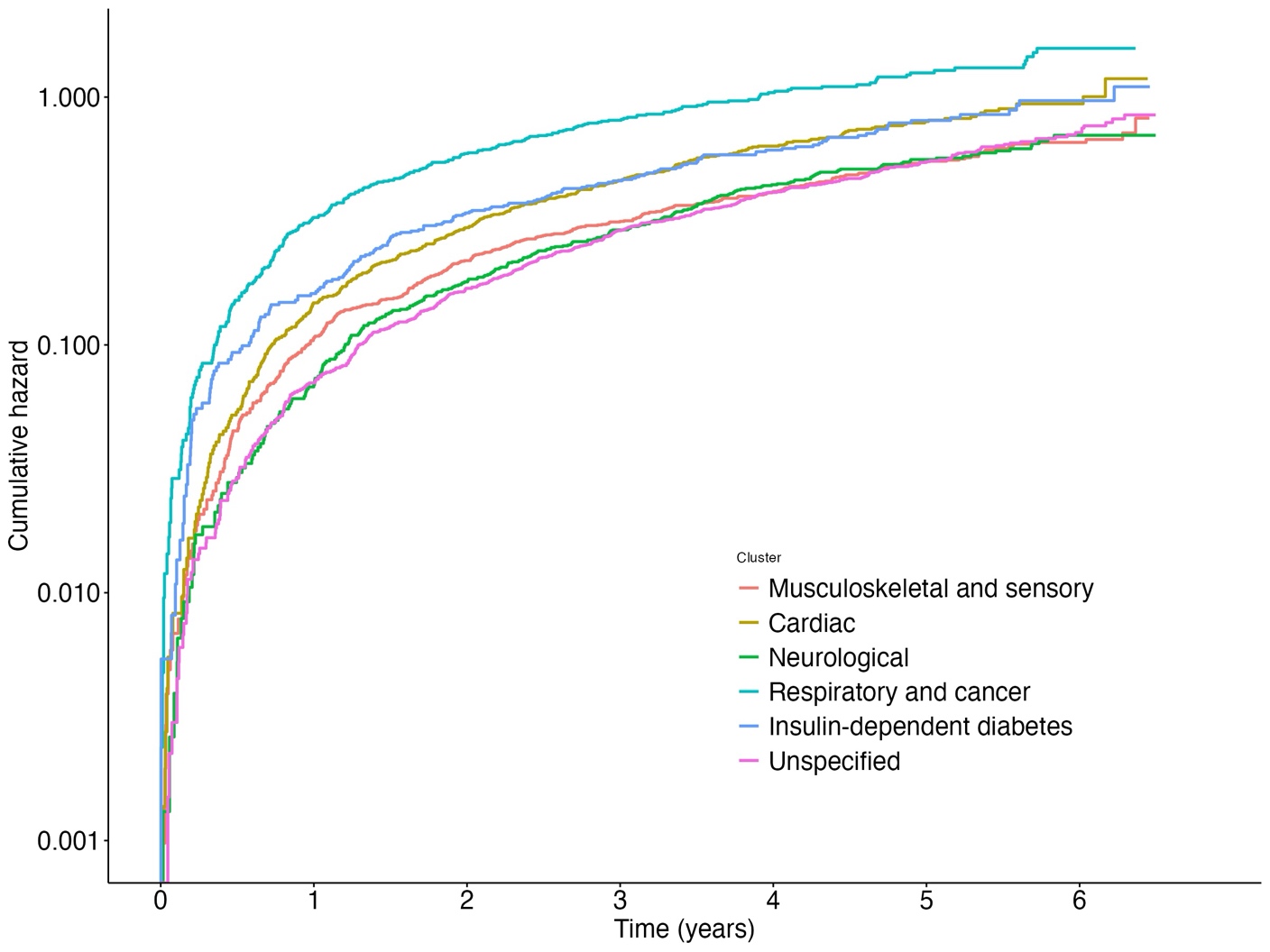
**

**Supplementary Figure S4. Obtained survey data for care-need certification, medical claims data and insurance registration data in Tsukuba City**

**
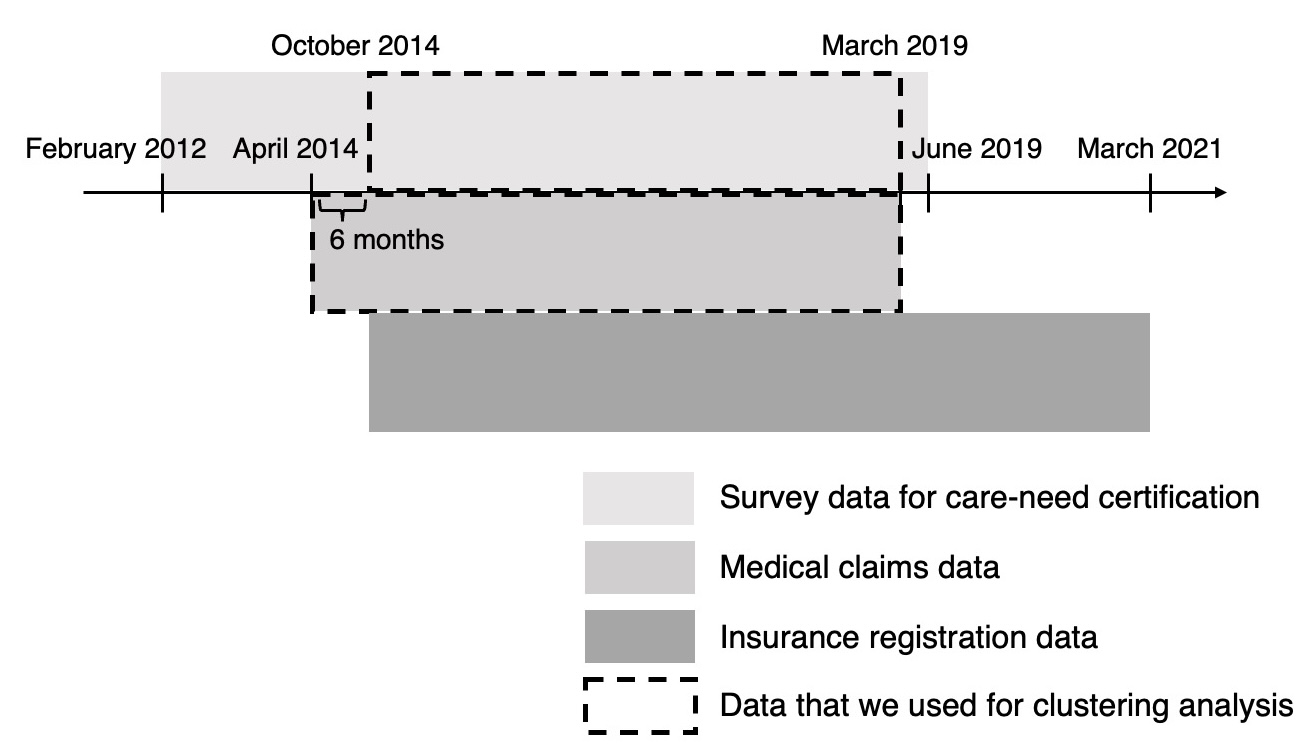
**

**Supplementary Table S1. The Xie-Beni index of each condition in a fuzzy c-means clustering algorithm**

| **Number of clusters** | **m parameter** | |
| --- | --- | --- |
|  | **m=1.1** | **m=1.5** |
| 2 | 0.0001898194 | 0.0002364669 |
| 3 | 0.0002419844 | 0.0002338178 |
| 4 | 0.0001900166 | 0.0001791536 |
| 5 | 0.0001594791 | 0.0001383843 |
| 6 | 0.0001364424 | 0.0002164881 |
| 7 | 0.0001865748 | 0.000201598 |
| 8 | 0.0001731017 | 0.0002095857 |

Footnote: Xie-Beni index indicates the optimal number of clusters when the value is the lowest.

**Supplementary Table S2. Definition of 22 diseases based on the International Classification of Diseases 10th Revision (ICD-10) codes**

| **Medical diagnosis category** | **ICD-10 codes** |
| --- | --- |
| Cerebrovascular diseases: |  |
| 1. Hemorrhagic stroke | I60–I62 |
| 1. Ischemic stroke | I63 |
| 1. Other cerebrovascular diseases* | I64–I69 |
| Cardiac diseases: |  |
| 1. Ischemic heart disease | I20–I25 |
| 1. Arrhythmia | I44, I45, I47–I49 |
| 1. Heart failure | I50 |
| 1. Other cardiac diseases | I01, I05–I09, I11, I13, I30–I43, I51, I52 |
| 1. Cancer | C00–C097 |
| Lower respiratory tract diseases |  |
| 1. Chronic obstructive pulmonary disease | J43, J44 |
| 1. Pneumonia | J12–18, J69 |
| 1. Other lower respiratory tract diseases | A15, A16, J20–J22, J40–J42, J45–J47, J60–J68, J70, J80–J86, J90–J94 |
| Joint diseases |  |
| 1. Rheumatoid arthritis | M05, M06 |
| 1. Other arthropathies | M00–03, M07, M10–M25 |
| 1. Dorsopathies (disorders of the back or spine) | M40–M54 |
| 1. Dementia | F00–F03, G30 |
| 1. Parkinson’s disease | G20 |
| Diabetes |  |
| 1. Insulin-dependent diabetes | E10–E14 with prescription records of insulin products** |
| 1. Non-insulin-dependent diabetes | E10–E14 with prescription records of oral antidiabetic drugs*** (without insulin products) |
| Visual or hearing impairment |  |
| 1. Visual impairment | H53, H54 |
| 1. Hearing impairment | H90, H91 |
| Fractures |  |
| 1. Femur fractures | S72 |
| 1. Other fractures | S02, S12, S22, S32, S42, S52, S62, S82, S92, T02, T08, T10, T12 |

ICD-10 = International Classification of Diseases 10th Revision.

*Including unspecified stroke and sequelae of cerebrovascular disease.

**Prescription records (WHO Anatomical Therapeutic Chemical classification ‘A10A’) were additionally used to define insulin-dependent diabetes.

***Prescription records (WHO Anatomical Therapeutic Chemical classification ‘A10B’) were additionally used to define Non-insulin-dependent diabetes.
